# Influence of surfactant-tailored Mn-doped ZnO nanoparticles on ROS production and DNA damage induced in murine fibroblast cells

**DOI:** 10.1038/s41598-020-74816-0

**Published:** 2020-10-22

**Authors:** Traian Popescu, Christien Oktaviani Matei, Ioana Dorina Vlaicu, Ioan Tivig, Andrei Cristian Kuncser, Mariana Stefan, Daniela Ghica, Luminita Claudia Miclea, Tudor Savopol, Daniela Cristina Culita, Mihaela Georgeta Moisescu

**Affiliations:** 1 National Institute of Materials Physics, Atomistilor 405A, 077125 Magurele, Romania; 2 Biophysics and Cellular Biotechnology Department, Excellence Centre for Research in Biophysics and Cellular Biotechnology, Carol Davila University of Medicine and Pharmacy, 8 Eroii Sanitari Blvd., 050474 Bucharest, Romania; 3 Ilie Murgulescu Institute of Physical Chemistry, Romanian Academy, 202 Splaiul Independentei, 060021 Bucharest, Romania

**Keywords:** Cell biology, Chemistry, Materials science

## Abstract

The present study concerns the in vitro oxidative stress responses of non-malignant murine cells exposed to surfactant-tailored ZnO nanoparticles (NPs) with distinct morphologies and different levels of manganese doping. Two series of Mn-doped ZnO NPs were obtained by coprecipitation synthesis method, in the presence of either polyvinylpyrrolidone (PVP) or sodium hexametaphosphate (SHMTP). The samples were investigated by powder X-ray Diffraction, Transmission Electron Microscopy, Fourier-Transform Infrared and Electron Paramagnetic Resonance spectroscopic methods, and N_2_ adsorption–desorption analysis. The observed surfactant-dependent effects concerned: i) particle size and morphology; ii) Mn-doping level; iii) specific surface area and porosity. The relationship between the surfactant dependent characteristics of the Mn-doped ZnO NPs and their in vitro toxicity was assessed by studying the cell viability, intracellular reactive oxygen species (ROS) generation, and DNA fragmentation in NIH3T3 fibroblast cells. The results indicated a positive correlation between the specific surface area and the magnitude of the induced toxicological effects and suggested that Mn-doping exerted a protective effect on cells by diminishing the pro-oxidative action associated with the increase in the specific BET area. The obtained results support the possibility to modulate the in vitro toxicity of ZnO nanomaterials by surfactant-controlled Mn-doping.

## Introduction

Engineered zinc oxide (ZnO) nanomaterials (NMs) possess unique physicochemical and optoelectronic properties which enable their extensive use in a large variety of applications in the technological (solar cells, photocatalysis, chemical sensors, spintronics)^1^, personal care and cosmetics (toothpaste, sunscreens, skin-care creams)^2^, food packaging^3^ and biomedical (antitumor, antimicrobial, antidiabetic, anti-inflammatory, drug delivery, bioimaging)^4–6^ sectors.

ZnO nanoparticles (NPs) have been shown to exhibit promising in vitro antitumor and antimicrobial activity based on the action of released Zn^2+^ ions and generated intracellular reactive oxygen species (ROS), leading in some cases to the activation of apoptotic signaling pathways in mammalian cells^7–9^. The antimicrobial effect of ZnO NPs was used as an active principle in the design of cosmetic and personal care products^10^ and functional textile fabrics^11,12^.

In vivo studies regarding the use of ZnO NPs as zinc sources or drug delivery systems in diabetes^13–15^ and cancer therapies^16^ have revealed their potential to interfere with zinc homeostasis and promote the bio-availability of therapeutic drugs or biomolecules (e.g., enhancing the efficiency of cancer therapy in animal models^17,18^, decreasing blood glucose by increasing the level of insulin in the blood of diabetic rats^13^).

Besides studying the potential of various ZnO-based nanostructures for therapeutics or other applications, extensive research has been dedicated to understanding the mechanisms of ZnO nanomaterials toxicity on non-malignant mammalian cells^19,20^. Although some common features were observed, like ZnO dissolution-dependent toxicity and intense oxidative stress responses, results obtained by different groups are difficult to compare and extrapolate due to the large variety of tested ZnO NMs, produced via different synthesis routes under various conditions and investigated using a diversity of methods on different cell lines^21^. However, in all cases, the primary synthesis-controllable characteristics that dictate the physicochemical and biological interactions of the studied ZnO NMs are particle size, shape, surface morphology, and doping. By controlling these parameters, one can, in principle, modulate both the toxicity and therapeutic activity of ZnO NMs.

The shape and size of nanomaterials obtained by wet chemical methods can be tailored by influencing their nucleation and growth phases. One efficient way to achieve this target is the use of structure-directing (or shape-control) agents (SDAs)^22^. SDAs are multifunctional organic compounds that induce constrained nanocrystal growth by promoting the growth of specific crystal facets while hindering others. In the case of ZnO, in the absence of SDAs, the polar nature of the ZnO nanocrystals favors the formation of 1-D structures by preferential growth along the c-axis. By using SDAs in the synthesis process, 3-D ZnO nanostructures can be obtained, ranging from spherical nanoparticles to structures with a higher degree of complexity^23^. Although some empirical theories on shape guiding and determination by SDAs are available^24^, the general action mechanisms of the shape-control agents are still poorly understood.

Besides modifications induced by SDAs, the size, morphology, and other important physicochemical properties of pure ZnO nanomaterials can be influenced by transition metal ions (TMIs) doping^25^. It has been shown that, among TMIs, manganese possesses good solubility in ZnO while maintaining its crystal structure and significantly affecting its particle shape and size as well as magnetic and optical properties^26^. Our group has recently reported results regarding the possibility to influence/tailor the particle morphology and dopant distribution in ZnO nanopowders doped with Mn by tuning the pH or thermal treatment conditions during the synthesis process^27,28^.

Besides being a convenient dopant for tuning the physicochemical properties of ZnO nanomaterials, manganese is also an essential bio-element. It is necessary for biochemical processes involving Mn-dependent enzymes (oxidoreductases, isomerases, hydrolases, transferases, and others) and Mn-metalloenzymes (phosphoenolpyruvate decarboxylase, arginase, glutamine synthetase, Mn superoxide dismutase) as well as for the normal metabolic activity of proteins, lipids, and carbohydrates^29^.

The influence of Mn-doping on the cytotoxicity and genotoxicity exhibited by ZnO NMs on non-malignant mammalian cells through oxidative stress responses was little studied and the specific mechanisms responsible for the observed effects remain unclear.

Guo et al*.*, 2016, have studied the effects of undoped ZnO NPs on murine photoreceptor cells and have shown that they inhibit the expression of manganese superoxide dismutase (MnSOD)^30^, which is “the major ROS detoxifying enzyme of cells”^31^. Yin and Casey^32^ showed that Mn-doped ZnO NPs induced the generation of higher intracellular ROS levels, associated with enhanced cytotoxicity, in comparison with undoped ZnO, although both released similar amounts of free Zn ions.

Since the doping process is strongly connected to the nanocrystal nucleation and growth phases, which are constrained in the presence of SDAs during NMs synthesis, we hypothesize that the Mn-doping of ZnO nanomaterials, and consequently their in vitro toxicity, can be directly influenced by the action of SDAs. At present, there is no consistent understanding of the way in which cytotoxicity is influenced by the SDA properties of the surfactants used during NMs synthesis.

In this context, the goal of our study was to assess the feasibility of influencing the Mn-doping of ZnO nanoparticles by SDAs/surfactant-induced constrained nanocrystal growth and study the influence of morpho-structural properties and doping concentration on the in vitro oxidative stress responses of the non-malignant cells treated with nanosized Mn-doped ZnO.

For this purpose, Mn-doped ZnO nanomaterials with different dopant concentrations were synthesized in the presence of either polyvinylpyrrolidone (PVP) or sodium hexametaphosphate (SHMTP). PVP is a biocompatible non-ionic polymer with reducing properties, frequently used in nanomaterials synthesis as a surface stabilizer and dispersant, and also one of the most studied SDAs^33^. SHMTP is a polyphosphate surfactant with good biocompatibility and dispersing properties, interesting for influencing the characteristics of ZnO nanomaterials due to the known interaction between ZnO and phosphate ions^34,35^. Only limited information is available regarding the effects of SHMTP on the doping of ZnO nanocrystals^36^.

The Mn-doped ZnO nanomaterials obtained in the presence of PVP and SHMTP were compared with respect to the relationship between their structural, morphological and doping characteristics and the viability changes, intracellular ROS generation, and DNA fragmentation induced in non-malignant murine fibroblast cells.

Moreover, we aimed to establish whether structure-directing agents may represent a non-expensive and facile way to engineer doping processes and modulate the biocompatibility of nanomaterials and to gain new insight regarding the relationship between Mn-doping level and ZnO in vitro toxicity.

## Experimental

### Materials synthesis

Zinc oxide powders doped with variable amounts of manganese ions were synthesized using a simple and cost-effective co-precipitation method. The following precursors have been used: zinc(II) nitrate hexahydrate [Zn(NO_3_)_2_·6H_2_O, purum p.a., > 99.0%, Sigma Aldrich], manganese(II) nitrate hydrate [Mn(NO_3_)_2_·xH_2_O, 99.98% (metals basis), Alfa Aesar], sodium hydroxide [NaOH, puriss p.a., ACS reagent, > 98%, HoneyWell/Fluka], polyvinylpyrrolidone [PVP, Sigma Aldrich], sodium hexametaphosphate [SHMTP, 96%, Aldrich]. The latter two were used as surface-active agents expected to influence the size and morphology of the produced ZnO particles. The synthesis procedure consisted of the following steps: zinc nitrate (0.0356 mol) and manganese nitrate (1.06; 10.6; and 42.4 µmoles) were added to the surface agent solution (PVP or SHMTP) heated at 60 °C and stirred for 30 min. Zinc nitrate to manganese nitrate ratio was varied in order to have nominal concentrations of Mn^2+^ ions of 50, 500, and 2000 ppm. The solution containing the surface agent and (Zn + Mn) ions was precipitated with a NaOH solution (0.024 mol) added dropwise under continuous stirring at 60 °C. The resulting suspension was allowed to age at a constant 60 °C temperature for several hours. The precipitates were separated by centrifugation, washed several times with double-distilled water, and let to dry overnight in an oven at 60 °C.

The samples prepared using the above-mentioned procedure are further labeled in the text as follows:

- For ZnO NPs prepared in the presence of PVP surfactant and with variable Mn^2+^ nominal concentrations: 50, 500 and 2000 ppm, the labels are ZOM50P, ZOM500P, and ZOM2000P, respectively;
- For ZnO NPs prepared in the presence of SHMTP surfactant and with variable Mn^2+^ nominal concentrations: 50, 500 and 2000 ppm, the labels are ZOM50S, ZOM500S, and ZOM2000S, respectively.
- The overall series of ZnO NPs prepared in the presence of PVP is denoted: ZOMP;
- The overall series of ZnO NPs prepared in the presence of SHMTP is denoted: ZOMS.

### Experimental techniques

Powder X-ray Diffraction (XRD) measurements were performed using a D8 ADVANCE diffractometer (BRUKER-AXS GmbH, Germany) with Ni filtered Cu radiation (λ = 1.54184 Å). The lattice parameters and the average crystallite size were determined by Rietveld refinement, using the Topas v.3 software (Bruker)^27^.

Fourier-Transform InfraRed (FTIR) spectroscopy measurements were performed using a Spectrum BX II (Perkin Elmer) spectrometer in the 4000–350 cm^−1^ spectral range, by accumulating 64 scans at a resolution of 4 cm^−1^. The samples were finely crushed with KBr in a 1:50 mass ratio and pressed into thin pellets^27^.

Transmission Electron Microscopy (TEM) studies were performed using a JEOL 2100 system equipped with an Energy Dispersive X-Ray Spectroscopy (EDS) detector and ASTAR crystallographic analysis unit. All the samples were prepared using the conventional powder method.

Electron Paramagnetic Resonance (EPR) spectroscopy investigations in the X (9.86 GHz) frequency band were carried out at room temperature on a Bruker ELEXSYS E-580 spectrometer equipped with the calibrated Super High QE (SHQE) cylindrical cavity resonator (ER 4123SHQE). Reference-free determinations of the Mn^2+^ ions concentration in weighted amounts of powder samples inserted into pure fused silica tubes were performed using the absolute spin quantitation routine included in the XEPR software from Bruker^28^.

BET surface area & Porosity measurements Nitrogen sorption isotherms were recorded at 77 K using a Micromeritics ASAP 2020 apparatus. Each sample was degassed at 100 °C for 12 h under vacuum before analysis. The BET surface area was calculated according to the Brunauer–Emmett–Teller (BET) equation, using adsorption data in the relative pressure range between 0.05 and 0.30. The total pore volume was estimated from the amount adsorbed at the relative pressure of 0.99. The pore size distribution curves were obtained using the Barrett-Joyner-Halenda (BJH) method from the desorption branch^46–48^.

## Results

### XRD investigations

**Figure 1:**
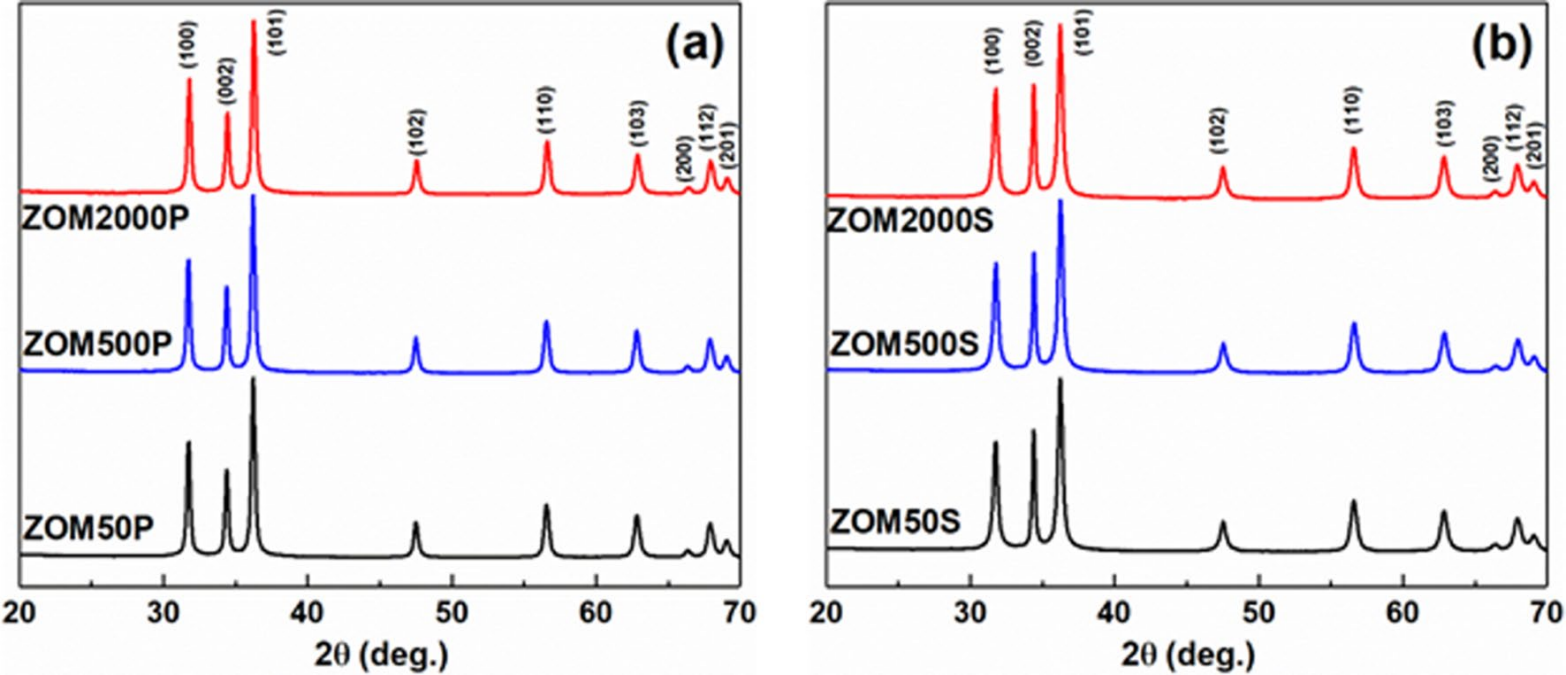
X-ray diffractograms of the ZnO:Mn samples prepared with: (**a**) PVP and (**b**) SHMTP.

Figure 1 presents the X-ray diffractograms of the two sets of ZnO samples doped with variable Mn^2+^ nominal concentrations in the presence of either PVP or SHMTP. The two sets of samples exhibit similar XRD patterns, indexed as single-phase ZnO with a hexagonal structure, space group P63mc (JCPDS 89-1397). In the case of the ZnO samples prepared in the presence of PVP (Fig. 1a), the relative intensities of the XRD peaks are typical for nanocrystals with polyhedral morphology, the average crystallite size being 38 ± 3 nm. On the contrary, for the ZnO samples prepared in the presence of SHMTP (Fig. 1b), a notable (002) texture is observed in their XRD patterns. The calculated crystallite size along (002) is 49 ± 4 nm, while for the other crystallographic directions it is 23 ± 2 nm. This result suggests that samples in the ZOMS series are expected to have a distinct morphology in comparison with the ZOMP samples due to the determined growth anisotropy.

The calculated lattice parameters (*a* = 3.2521 ± 0.0003 Å, *c* = 5.2107 ± 0.0003 Å) and the crystallite size are independent of the doping level within the experimental errors. The substitution of the Zn^2+^ ions with Mn^2+^ ions would produce a very slight expansion of the ZnO lattice, as the ionic radii of Zn^2+^ and Mn^2+^ in four-fold tetrahedral coordination are close to each other: 0.74 Å (Zn^2+^) and 0.80 Å (Mn^2+^)^38^. Therefore, as expected, a low doping level does not affect the lattice parameters.

### TEM investigations

TEM analysis revealed morphological and structural aspects of the investigated samples. Two types of systems have been identified, defined by the influence of the used surfactants: i) a system of quasi-spherical, monodispersed nanoparticles with a size of ~ 30 nm and ii) a system consisting of both single nanoparticles and broad distribution of aggregates with complex morphologies, with a resulting size distribution spreading from ~ 30 nm to ~ 150 nm, in the PVP and SHMTP samples, respectively.

**Figure 2:**
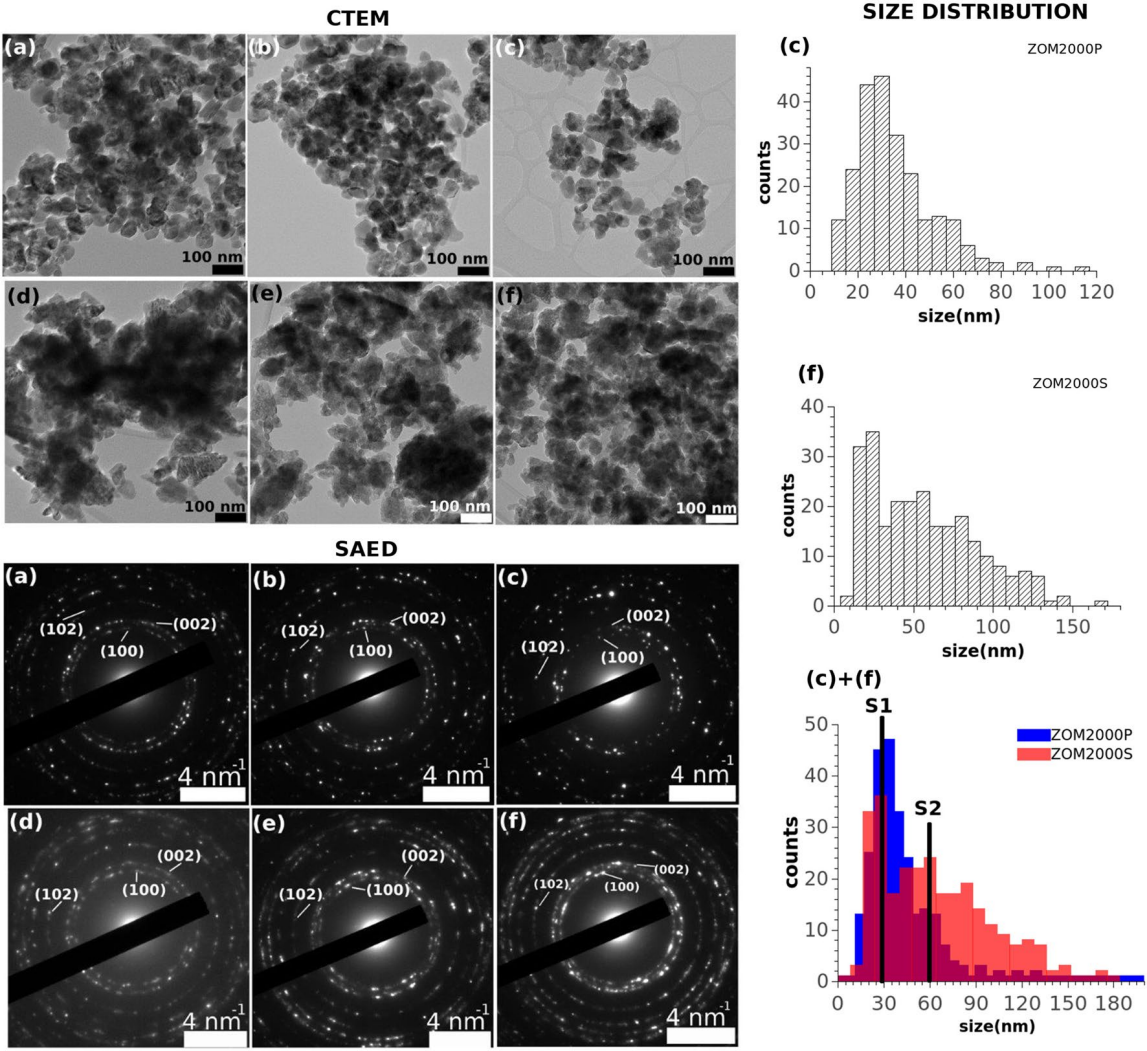
CTEM (top left), SAED (bottom left) and two size distributions (right) of: ZOM50P (**a**), ZOM500P (**b**), ZOM2000P (**c**), ZOM50S (**d**), ZOM500S (**e**), ZOM2000S (**f**).

The CTEM images in Fig. 2 (a)-(c) (top left) show the simple morphological system of the PVP samples whereas in Fig. 2 (d)-(f) (top left) the complex morphologies present in the SHMTP samples are pictured.

All the SAED patterns in Fig. 2 (bottom left) display lattice planes of hexagonal ZnO (space group 186). All the samples are well crystallized.

A quantitative description of the two types of morphologies of ZOM2000P and ZOM2000S, as observed by conventional imaging, is shown in the size distribution in Fig. 2 (right). The bi-modal behavior of the two distributions, with maxima at S1 = 30 nm and S2 = 60 nm as well as their corresponding FWHM, i.e. relatively narrow for S1 for both samples but much broader for S2 of ZOM2000S, suggests that in the presence of the PVP surfactant the NPs system tends to be much more uniform (mainly with small crystal sizes), whereas in the presence of SHMTP surfactant it exhibits a much more complex morphology (mainly with large crystal sizes). It is worth mentioning that both types of nanoparticles as they are quantitatively described by S1 and S2 are present in both ZOM2000S and ZOM2000P, but their prevalence changes dramatically.

### EPR spectroscopy

**Figure 3:**
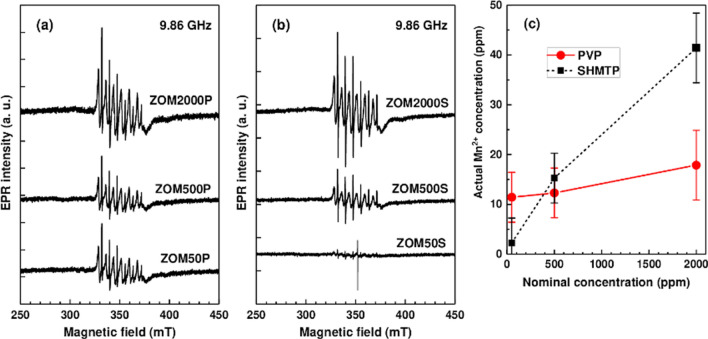
EPR spectra of the (**a**) PVP and (**b**) SHMTP ZnO:Mn samples, normalized by mass and spectrometer parameters; (**c**) Actual concentration of the Mn^2+^ ions in the ZnO:Mn samples with different nominal doping levels.

The EPR spectra of the two sets of ZnO:Mn samples, normalized for differences in mass and spectrometer parameters (i.e. resonator quality factor Q, number of scans), are displayed in Fig. 3 a**-**b. As expected, the intensity of the spectra increases with the increase in the Mn^2+^ doping levels. The spectra are quite alike for the two sets of samples prepared with different surfactants, except in the case of the ZOM50P sample for which the intensity of the spectrum is markedly higher than for the corresponding ZOM50S sample.

The dominant features in the EPR spectra are two groups of six lines with different intensities and linewidths (~ 0.3 mT and ~ 1.7 mT, respectively), characteristic to the hyperfine structure of isolated Mn^2+^ ions (*S* = 5/2, *I* = 5/2). The similar line separation of ~ 7.8 mT shows that both sets of lines belong to Mn^2+^ ions localized in the ZnO lattice, namely in ZnO nanocrystals (the broad lines) and in a disordered ZnO phase (the narrow lines)^39^. The actual concentrations of the Mn^2+^ ions in weighted amounts of the investigated ZnO:Mn samples were determined by the quantitation of the recorded X-band EPR spectra. The error in these determinations was estimated to decrease from 30% for the 50 ppm nominal concentration to 25% for 2000 ppm. According to the results presented in Fig. 3c, the actual Mn^2+^concentration is one to two orders of magnitude lower than the nominal doping concentration. Moreover, the actual concentration of the Mn^2+^ ions in the PVP samples is lower than in the SHMTP samples with the same nominal concentration, except in the case of the ZOM50P sample. Another oddity of the ZOM50P sample is that the actual Mn^2+^ concentration is very close to the one determined for the ZOM500P sample.

### FTIR spectroscopy

**Figure 4:**
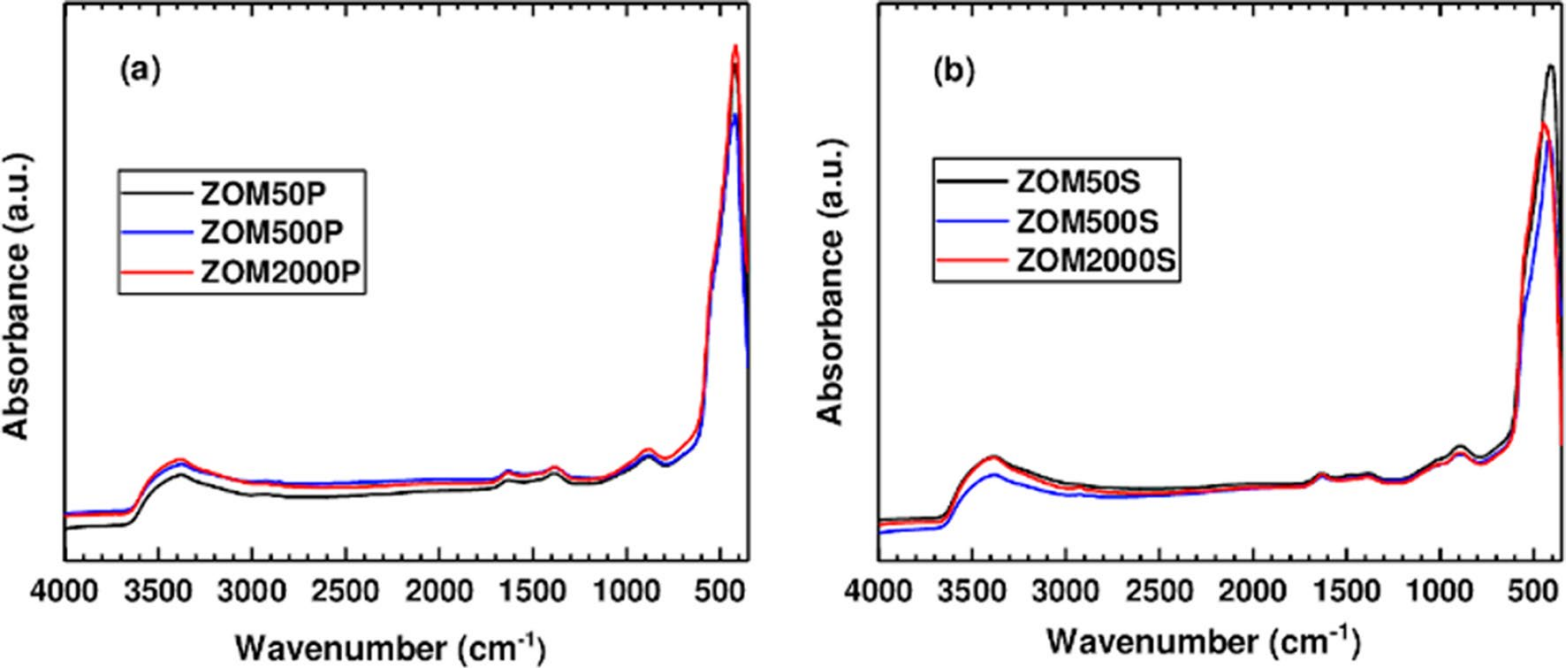
Infrared spectra of the ZnO:Mn samples prepared in the presence of: (**a**) PVP and (**b**) SHMTP.

The composition and the molecular structure of the samples were analyzed by FTIR spectroscopy, using the KBr method at RT, as shown in Fig. 4. Both series of samples were measured and the influence of the doping level on their spectroscopic features was evaluated. Another investigated aspect was the presence of remaining surfactant in the samples. Both series of samples, for all three Mn nominal concentrations, displayed the same absorption bands in the 3500–750 cm^−1^ spectral range. The broad absorption band observed around 3500 cm^−1^ corresponds to the stretching vibration of the O–H group from water molecules. The weak absorption band at ~ 1630 cm^−1^ is assigned to the deformation vibration of the water molecules adsorbed on the surface of the nanoparticles. The two weak absorption bands observed in the 1400–750 cm^−1^ spectral range are attributed to NO_3_ groups from residual precursors used in the synthesis process^27,28^.

For the ZnO:Mn samples prepared in the presence of PVP (Fig. 4a), the absorption maximum at 420 cm^−1^ assigned to the Zn–O vibration band is independent of the Mn nominal concentration. This observation is in agreement with the XRD and TEM observations regarding the morphological aspects of the ZnO NPs (PVP) which do not change with the Mn nominal concentration. Previous FTIR investigations reported this absorption maximum for ZnO particles with spherical morphology^40,41^. This observation is in agreement with our XRD and TEM results. On the contrary, the ZnO:Mn samples prepared in the presence of SHMTP (Fig. 4b) present blue-shifted Zn–O absorption bands dependent on the Mn nominal concentration. Thus, for 50 ppm a peak position at 410 cm^−1^ was observed for the Zn–O vibration, which indicates a different morphology than for the samples prepared in the presence of PVP. For the SHMTP samples prepared with 500 ppm and 2000 ppm nominal concentration, the absorption maximum for the Zn–O vibration is observed at 420 cm^−1^ and 440 cm^−1^, respectively. This change in the peak position of the Zn–O absorption bands is due to the perturbation of the Zn–O-Zn network by the presence of the embedded Mn atoms^42^, which may lead to the morpho-structural changes observed also in TEM. We could not identify any absorption bands corresponding to the vibrations of the chemical bonds present in the surfactants^43–45^ in the FTIR spectrum of any of the six samples. The samples can thus be considered surfactant-free. The efficient washing off of the surfactants from the ZnO:Mn samples is very important, since, in a recent review, Naveed Ul Haq et al*.*^21^ reported that ZnO NPs covered with different surfactants possess varied and increased toxicity which is affecting the environment, the animals and even the humans.

### BET & porosity

**Figure 5:**
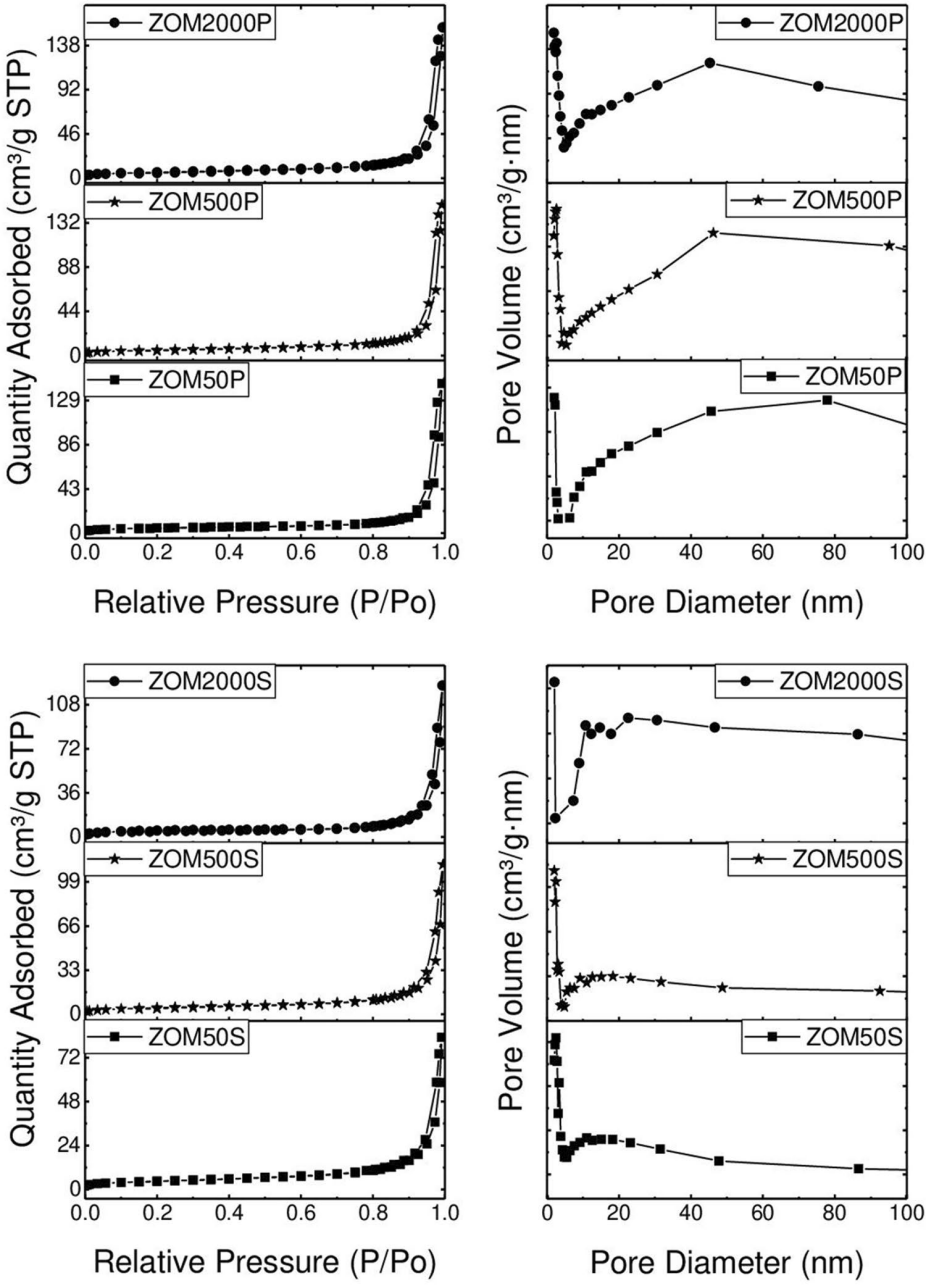
N_2_ adsorption–desorption isotherms (left) and pore size distribution curves (right) of the samples.

The recorded N_2_ adsorption–desorption isotherms are shown in Fig. 5. All the samples display typical type IV isotherms accompanied by H3 type hysteresis loops, according to the IUPAC classification^46^. The hysteresis loops appear at high relative pressures (> 0.8), which suggests the presence of non-rigid aggregates with irregular and large slit-shaped pores^47,48^. The pore size distribution curves (Fig. 5, right side) confirm these observations and the data are in good agreement with the microscopic investigations that reveal irregular pores randomly distributed, mainly consisting of interparticle voids.

The textural parameters (Table 1) present low values, as expected when taking into account the morphology of the samples. It can be noticed that the S_BET_ and total pore volume values are slightly higher for the sample obtained using PVP as a surface agent (surfactant) in comparison with the samples prepared in the presence of SHMTP. The values are directly proportional to the dopant concentration, regardless of the surface agent (surfactant) used. This can be attributed to the formation of larger aggregates of nanoparticles in the case of the samples prepared in the presence of SHMTP, as TEM investigations revealed. These results are in agreement with the size distributions obtained from TEM determinations, the ZOMP samples with small particle size and narrow size distribution being associated to larger surface areas and larger pore volumes compared to the ZOMS samples partially composed of large size aggregates with broad size distribution, characterized by reduced surface areas and smaller pore volumes.

**Table 1:** Textural parameters of the samples.

Sample	S_BET_ (m^2^ g^−1^)	Pore volume (cm^3^ g^−1^)
ZOM50P	17.4	0.224
ZOM500P	18.8	0.231
ZOM2000P	21.1	0.242
ZOM50S	15.9	0.128
ZOM500S	17.2	0.173
ZOM2000S	18.4	0.190

### Cellular studies results

#### Cell viability tests

**Figure 6:**
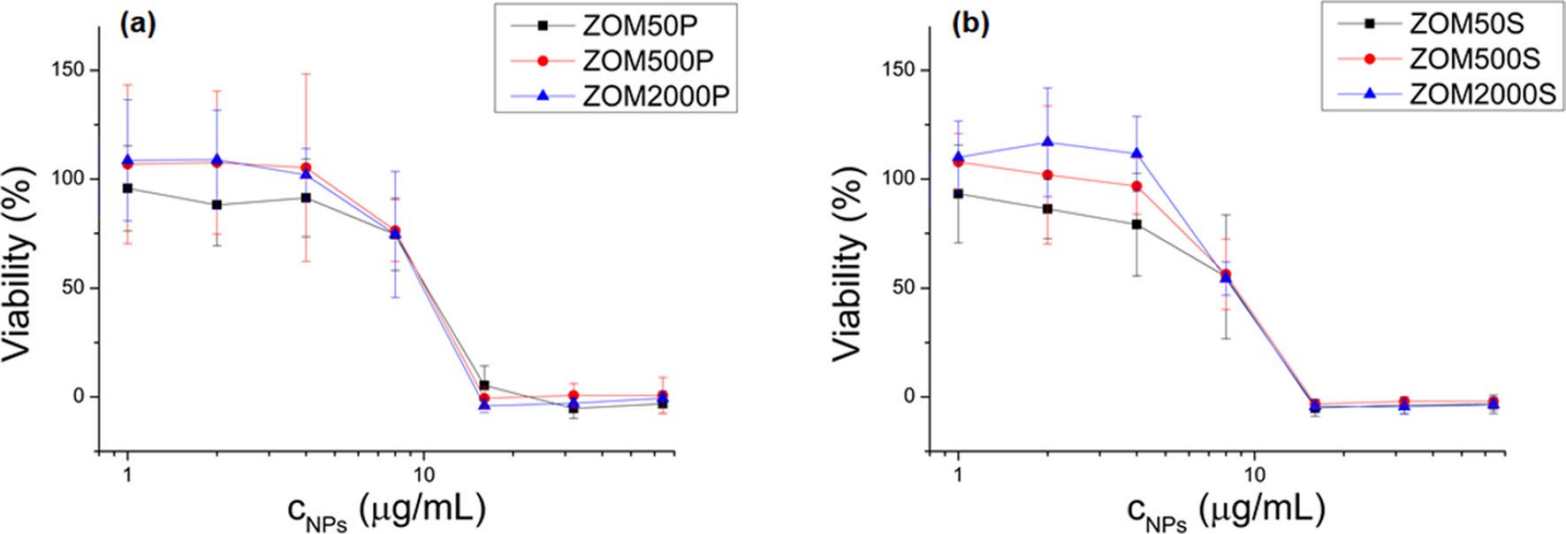
MTS viability tests of NIH3T3 cells treated with ZnO NPs doped with variable Mn^2+^ nominal concentrations (50, 500 and 2000 ppm) at different NPs concentrations (1, 2, 4, 8, 16, 32, and 64 µg/mL): (**a**) NPs prepared with PVP; (**b**) NPs prepared with SHMTP. All values were normalized to the control (0 µg/mL NPs).

For NPs concentrations below 4 µg/mL, the cell viability is not significantly affected (see Fig. 6); for nominal doping concentrations of 500 ppm and 2000 ppm of Mn^2+^, a slight growing stimulation effect (viabilities above 100%, more pronounced in the case of ZOM2000S) can be observed.

At these NPs concentrations, in the case of the 50 ppm Mn^2+^ nominal concentration (black lines in Fig. 6), the viability slightly decreases with NPs concentration (even if not statistically significant). At higher NPs concentrations, the viability drops, going down to zero for concentrations above 16 µg/mL.

#### Intracellular ROS measurement

**Figure 7:**
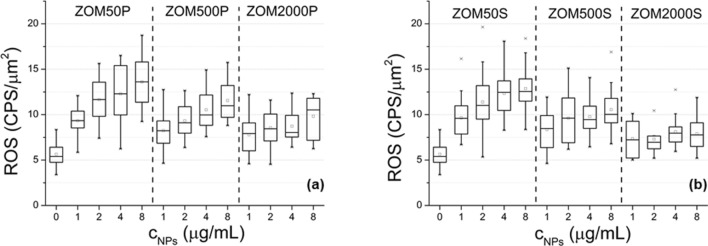
ROS production of NIH3T3 cells treated with different concentrations of ZnO NPs doped with 50, 500 and 2000 ppm Mn^2+^: (**a**) NPs prepared in PVP; (**b**) NPs prepared in SHMTP.

The ROS production increases with the concentration of NPs in both PVP and SHMTP formulations (Fig. 7). The higher the nominal doping concentration, the less pronounced is the dependency of the ROS production on the NPs concentration: the doping concentration flattens the dependency of ROS production on NPs concentration. This behavior was observed to be more pronounced in the case of ZOMS samples compared to ZOMP. For ZOM2000S, the dependence of the ROS production on the NPs concentration becomes insignificant.

#### Cellular DNA fragmentation

**Figure 8:**
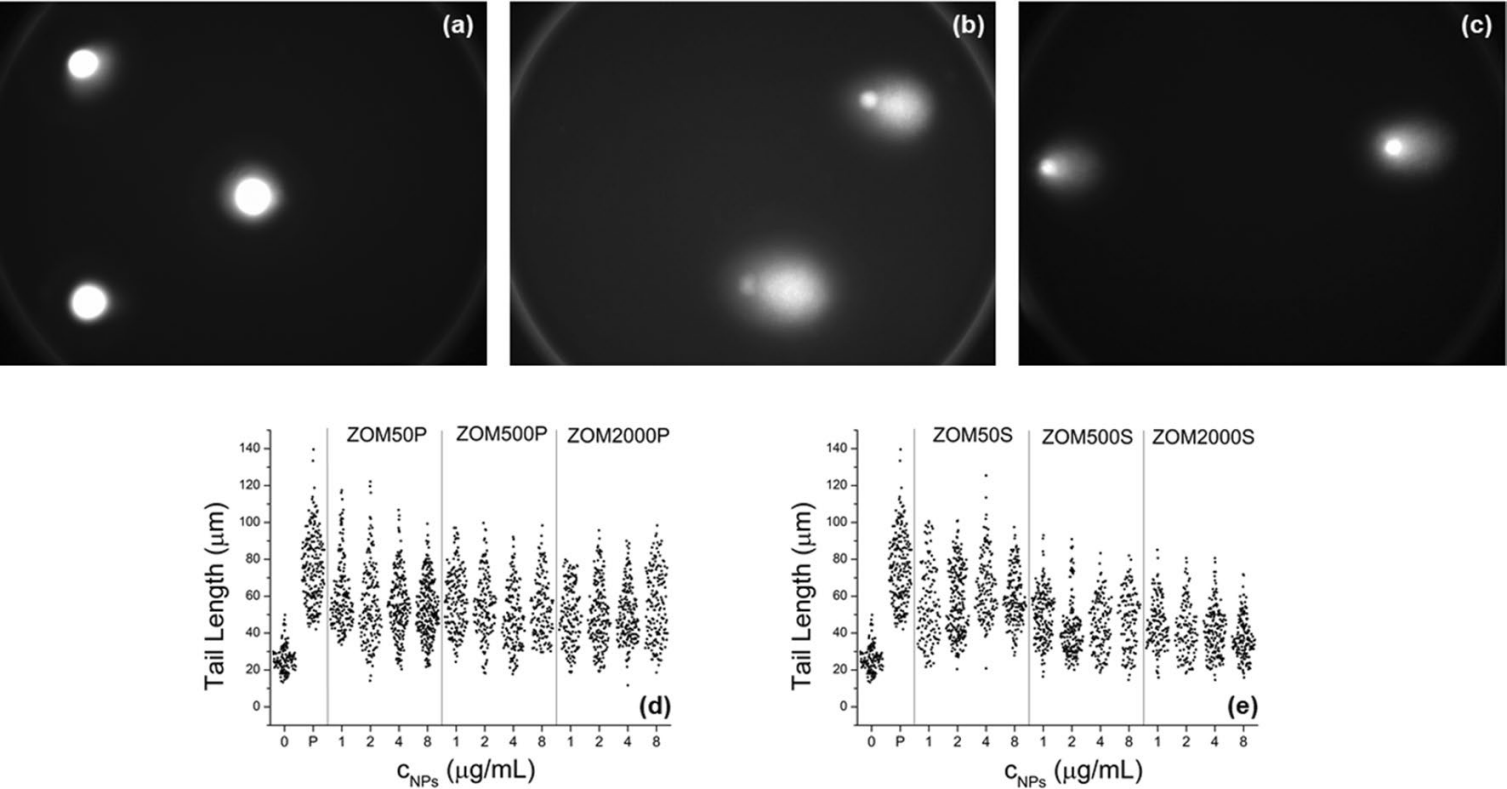
Comet assay fluorescence images (objective 40x): (**a)** control cells; (**b)** cells exposed to ZOM50P NPs (4 µg/mL); (**c)** cells exposed to ZOM50S NPs (4 µg/mL). Tail lengths of comets obtained from NIH3T3 cells treated with different concentrations of ZnO NPs doped with 50, 500 and 2000 ppm Mn^2+^: (**d)** NPs prepared in PVP; (**e)** NPs prepared in SHMTP. “0” represents the negative control (cells untreated with NPs) and “P” represents the positive control (cells treated with H_2_O_2_).

In order to assess the DNA fragmentation, comet assay images were acquired for untreated cells and cells treated with ZOMP and ZOMS NPs, respectively, as exemplified in Fig. 8 a-c. In the case of the cells exposed to NPs, the fragmentation of DNA was revealed by the presence of the comet-shaped fluorescent halo shown in Fig. 8b and c. No such halo was observed in the case of control cells.

The tail lengths of the comets are presented in Fig. 8d and e, for the PVP and SHMTP formulations, respectively.

For all NPs concentrations DNA fragmentation was observed (tail lengths from exposed cells are significantly longer compared to the negative control). The DNA fragmentation induced by exposing cells to NPs was not as thorough as in the case of the positive control (cells treated with H_2_O_2_).

In the case of the PVP formulation (Fig. 8d), increasing either the NPs or Mn nominal doping concentrations does not produce a further augmentation of the DNA fragmentation.

In the case of the SHMTP formulation (Fig. 8e) the following observations can be made:

- Increasing the Mn^2+^ nominal doping concentration reduces the DNA fragmentation (by comparing it with ZOM50S, ZOM500S, and ZOM2000S).
- For each Mn^2+^ nominal concentration group, the DNA fragmentation does not vary with increasing the NPs concentration, with the remarkable exception of ZnO NPs doped with 2000 ppm Mn^2+^, where increasing the concentration of NPs significantly reduces the DNA fragmentation.

## Discussion

Two series of Mn-doped ZnO NPs, each with a nominal dopant concentration of 50, 500, and 2000 ppm, were obtained following a surfactant-assisted co-precipitation synthesis method, in the presence of either PVP or SHMTP. In order to assess the influence of the used surfactants/SDAs on the physicochemical properties of the produced nanomaterials, the synthesized samples were characterized with respect to their crystal structure, particle size and morphology, textural properties (specific surface area and porosity and surface chemistry) and Mn-doping efficiency. The relationship between the observed surfactant-controlled properties of the synthesized NMs and their in vitro oxidative stress and cytotoxic effects was investigated by evaluating the cellular viability, intracellular ROS generation, and DNA fragmentation induced in non-malignant murine fibroblast NIH3T3 cells.

In the case of the ZOMP samples, the shape-directing action of PVP suppressed the c-direction of the ZnO crystals and led to the growth of single phase quasi-spherical monodisperse nanoparticles (≈ 38 nm), with lattice parameters unaffected by the doping process. The difference in the particles morphology suggested by the XRD results is confirmed by TEM investigations. The constancy of the lattice parameters with respect to the changing nominal concentration of Mn^2+^ is not surprising considering the low real concentration of Mn^2+^ ions incorporated in the ZOMP samples (12–20 ppm as indicated by the EPR results illustrated in Fig. 3c) and the small difference between the ionic radii of Zn^2+^ and Mn^2+^. It thus appears that the nucleation and growth of the ZOMP nanocrystals were mainly dictated by the action of PVP which hindered the incorporation of Mn into the samples.

The ZOMP nanoparticles exhibit type IV N_2_ adsorption–desorption isotherms (Fig. 5 (left)) which are characteristic for mesoporous materials. Moreover, the pore size distribution (Fig. 5 (right)) and the fact that the hysteresis loops appear at high relative pressures indicate the presence of non-rigid particle aggregates, in agreement to the TEM results. The values of BET specific surface area and total pore volume (Table 1) are directly proportional to the dopant concentration. Considering the low level of doping, it appears that these surface properties are very sensitive to the presence of Mn^2+^ ions. The low measured values of the BET surface area are not surprising considering the low synthesis temperature and the lack of thermal treatment after co-precipitation.

In comparison to ZOMP, the ZOMS samples exhibit significant differences regarding particle size distribution, morphology, and doping efficiency. The samples prepared in the presence of SHMTP consist of anisotropic ZnO NPs and aggregates with complex morphologies, leading to a broad size distribution ranging from ~ 40 nm to ~ 150 nm. The notable (002)-shape anisotropy, with a crystallite size of 49 ± 4 nm along (002) and 23 ± 2 nm for other crystallographic directions, indicates that the particle extension is promoted in the c-direction. SHMTP is thus less effective in constraining the shape of the ZnO nanocrystals in comparison to PVP. The anisotropic growth appears to facilitate the incorporation of Mn^2+^ ions into the ZOMS samples, a concentration of 42 ppm of Mn^2+^ being found by EPR determinations in the ZOM2000S sample (Fig. 3c). The increased doping efficiency in the ZOMS samples compared to the ZOMP samples could be due to their particular morphology, which exposes crystal faces with favorable binding energy for the Mn^2+^ ions^49^. The surprisingly low amount of Mn detected by EPR in both ZOMP and ZOMS series could be due to either inefficient doping due to the synthesis conditions or the presence of larger amounts of Mn in the samples in the form of the EPR silent Mn^3+^ ions. Concerning the textural properties of ZOMS, the measured BET specific areas and total pore volume are directly proportional to dopant concentration but the S_BET_ values are smaller compared to those of the ZOMP samples (Table 1). Similar to the case of ZOMP, the type IV N_2_ adsorption–desorption isotherms of ZOMS (Fig. 5 (left)) indicate the mesoporous nature of the samples. The pore size distribution (Fig. 5 (right)) together with the high values of the relative pressure where hysteresis loops occur suggest the presence of non-rigid aggregates, larger than in the case of the ZOMP series, with large slit-shaped irregular pores.

The FTIR spectroscopy investigations (Fig. 4) confirmed that no trace of the two surfactants remained in any of the studied ZnO:Mn samples after the washing and drying processes, any direct involvement of the surfactants in the in vitro action of the tested nanomaterials being thus excluded.

To summarize, the two series of samples, ZOMP and ZOMS, are distinct with respect to three aspects: i) particle size and morphology; ii) Mn-doping level; and, iii) specific surface area and porosity. While PVP induced the formation of quasi-spherical monodisperse nanoparticles, SHMTP allowed anisotropic growth on the c-direction leading to complex aggregates with a large size distribution. The Mn-doping of the synthesized ZnO nanocrystals was hindered by PVP and facilitated by SHMTP. Also, PVP led to higher specific surface areas and larger pore volumes compared to SHMTP. These results suggest that PVP could be helpful in producing mesoporous ZnO nanomaterials with high surface area and large pore volume for photocatalysis or gas sensing applications while SHMTP could be employed when Mn-doped anisotropic ZnO nanoparticles are desired.

Regarding the in vitro toxicity effects of the studied Mn-doped ZnO NPs, the MTS viability tests allowed the determination of the NPs concentrations for which it was justified to perform the other types of studies (ROS production and DNA fragmentation). NPs concentrations of up to 4 µg/mL did not significantly impact cellular growth. At these low NPs concentrations, the MTS assay showed that increasing the Mn^2+^ doping level diminishes the cytotoxic effect of NPs for both surfactant formulations.

The ROS production and DNA fragmentation revealed the oxidative stress induced in the cells exposed to NPs. The results of both tests led to the conclusion that oxidative stress is lower when using higher Mn-doping, this tendency being more pronounced in the case of the SHMTP formulation. For instance, the protective effect of doping is better observed in the case of ZOM2000S, where the ROS production does not depend on the NPs concentration anymore and the DNA fragmentation was found to be the smallest.

As it was shown by Laurent et al*.*, 2005, treating NIH3T3 fibroblasts with low amounts of H_2_O_2_ increased their proliferative rate, while further increased amounts of H_2_O_2_ resulted in growth arrest and cell death^50^. In our case, the ROS production due to small concentrations of NPs seems to play a similar role: at low NPs concentrations, there is a slight increase in the cell viability compared to controls, while at high NPs concentrations the viability drops. At higher NPs concentrations, the higher amount of ROS production exceeds the defense capability of the cells. Doping with Mn^2+^ has an antioxidant protective effect which is more pronounced in the case of the SHMTP series, probably due to the higher Mn^2+^ nominal concentration (see Fig. 6) in samples with the same NPs amount.

It is established that one of the main mechanisms responsible for the in vitro toxic effects of the ZnO nanomaterials involves the action of the Zn^2+^ ions released due to ZnO dissolution in alkaline phosphate media^19^. Moreover, it has been shown by several groups that the solubility of the ZnO nanoparticles in such media is diminished by TMIs doping^32,51^. Based on these considerations, we argue that the observed negative correlation between the cytotoxic and oxidative stress responses, on the one hand, and the doping level of the tested Mn-doped ZnO NMs from each series (ZOMP or ZOMS), on the other hand, is caused by the reduction of the ZnO dissolution induced by Mn-doping. This argument is also supported by the observed weakening of the dependence of the magnitude of the oxidative stress indicators on the concentration of the tested NPs with increasing dopant concentration in either ZOMP or ZOMS.

A comparison between the two series with respect to the dopant-dependent dissolution aspect would be forced since the dissolution of ZnO (and other types of nanoparticles) is also known to be size- and shape-dependent^52,53^.

Previous ZnO toxicology studies considered that the specific surface area is one of the key factors involved in the toxicity of ZnO nanomaterials^54^. In the case of ZOMP samples, the interaction of their larger surfaces with the cells and culture environment led to enhanced intracellular ROS production and DNA damage in comparison with the ZOMS series which was associated to smaller surface areas. This result indicates a positive correlation between the specific surface area and the magnitude of the studied in vitro toxicological effects. Such a relationship is expected considering the higher cellular stress experienced by cells exposed to larger nanomaterial surfaces^55^. This finding can also be described in terms of particle size distributions, emphasizing that samples with small particle size and narrow size distributions (e.g. ZOM2000P in Fig. 2), associated with bigger **S**_**BET**_ values, induce more intense cell responses in comparison to samples with larger particles/aggregate size and broad size distribution (e.g. ZOM2000S in Fig. 2) which are characterized by smaller surface areas. However, in each of the studied sample series, ZOMP and ZOMS, there is a negative correlation between the values of **S**_**BET**_ (which are directly proportional to the nominal doping concentration) and the cell oxidative responses. Based on these considerations, one can thus argue that Mn-doping exerts a protective effect on cells by diminishing the pro-oxidative action of the increased specific surface area.

The present results encourage the conduction of further studies on the use of surfactants/shape-directing agents to control the Mn-doping of ZnO and the mechanisms of in vitro toxicity of Mn-doped ZnO nanomaterials.

## Conclusions

Two surfactants with distinct shape-directing properties, polyvinylpyrrolidone (PVP) and sodium hexametaphosphate (SHMTP), were used in a low temperature co-precipitation synthesis method to produce quasi-spherical or anisotropic Mn-doped ZnO nanocrystals, respectively. The two types of nanomaterials showed differences with respect to size distribution, Mn-doping level and surface properties. The PVP-based synthesis led to ZnO samples consisting of nanoparticles with a mean size of ~ 38 nm, quasi-spherical morphology and monomodal size distribution. The ZnO samples obtained in the presence of SHMTP were composed of larger nanoparticles with broad size distribution, ranging from ~ 40 to 150 nm, and with complex morphologies.

Comparing the samples in terms of textural properties, the ZOMP samples present larger surface areas and larger pores than the ZOMS samples. The values of both textural parameters increased with Mn concentration in all cases.

In what regards the NPs doping, PVP was shown to hinder Mn incorporation into ZnO while SHMTP allowed for a more efficient Mn-doping.

The relation between relevant material properties, Mn-doping level, specific surface area and porosity, and the main in vitro oxidative stress toxic effects was assessed using murine fibroblast cells. The cell viability, intracellular ROS production, and DNA fragmentation were shown to depend on the morpho-structural properties of the tested ZnO nanoparticles as well as on the Mn-doping characteristics dictated by the used surfactants. The ZnO samples prepared in the presence of PVP were shown to be more cytotoxic than the ones prepared in the presence of SHMTP.

The ZnO PVP samples (ZOMP) display a stronger cytotoxic effect compared to SHMTP samples (ZOMS) due to morpho-structural characteristics that make them more reactive in the in vitro environment like smaller particle size, narrow size distribution, larger surface areas and pore volumes, and Mn incorporated inefficiently.

The cytotoxic action is diminished with increasing the Mn concentration as follows: the cell viability is affected more pronouncedly in the case of ZOMP samples but the effect weakens at higher doping levels; the cells exposed to ZOMP samples produce a higher quantity of ROS, which also decreases when the Mn concentration is increased; the DNA fragmentation is enhanced in the cells treated with ZOMP samples but it does not depend on the NPs concentration or Mn concentration. A peculiar effect was observed in the case of the ZOM2000S sample, for which the DNA fragmentation was reduced with increasing the NPs concentration.

The reduction of the cytotoxic effects following the increase of Mn-doping level suggests the possibility of using Mn-doping to improve the cytocompatibility of ZnO nanomaterials, even if they have characteristics, such as small particle size and large surface area, generally known to correlate to high cytotoxic effects.

Why are these findings important? When considering ZnO nanoparticles for antimicrobial applications, cosmetics or topical ointments for dermatological use, it is of prime importance to ensure their biocompatibility while maintaining the desired functional properties. Our study indicates that the use of convenient shape-directing agents and surfactants during the synthesis process and manganese doping may constitute a valid approach for this purpose.

Aside from gaining new insight regarding the ZnO in vitro toxicity modulation by surfactant-tailored morpho-structural properties and Mn-doping, our results suggest that structure-directing agents may represent a non-expensive and facile way to engineer doping processes and modulate the biocompatibility of nanomaterials.

## Methods

### Cellular studies

#### Cell cultures assay

Murine fibroblast NIH3T3 cells (ATCC CRL-1658) were cultured in high glucose Dulbecco’s Modified Eagle Media (Sigma D5796, USA) with 2 mM L-glutamine, supplemented with 10% Fetal Bovine Serum (Sigma F7524, USA), at 37 °C in humidified 5% CO_2_ atmosphere.

For cell viability tests, the cells were cultured in 96 well plates (TPP 92,196), for ROS measurements the cells were cultured in 9.2 cm^2^ Petri dishes (TPP 93,040), and for comet assay, the cells were cultured in 12 well plates (Greiner Cellstar 665,180). In each case, the cells were grown for 24 h prior to adding the NPs suspensions.

### Cell viability test

CellTiter 96 Aqueous One Solution Cell Proliferation Assay kit (Promega G3581, USA) (MTS) was used to measure the cell viability. Viable cells convert the tetrazolium reagent to the soluble formazan product, the concentration of which can be photometrically measured (maximum absorption at 490 nm). The formazan concentration is linearly correlated to the number of viable cells.

Sterile ZOMP/ZOMS NP suspensions were added in each well at final concentrations of 0, 1, 2, 4, 8, 16, 32, and 64 µg/mL (for each concentration four identical wells were used) and the cells were further incubated for 24 h; the medium was then removed and the cells were washed with 0.9% NaCl solution. 300 µL of Dulbecco’s Modified Eagle Media without Phenol-red (Gibco 21063-029, UK) containing MTS (volume ratio 5:1) was added to each well and incubated 2 h at 37 °C. The clear supernatant was transferred to new plates and the absorbance at 490 nm was recorded using a plate reader (Awareness Technology Inc., Taiwan). After subtracting the blank value, the absorbance values were normalized by dividing the average absorbances of identical samples by the average absorbance of the controls (samples with 0 µg/mL NPs).

### Intracellular ROS measurement

The Image-iT Live Green Reactive Oxygen Species Detection Kit (TermoFisher Scientific I36007, USA) (H_2_DCFDA) was used to measure the production of ROS induced by ZOMP/ZOMS. H_2_DCFDA is a membrane-permeant nonfluorescent compound. After entering the cell, it is deacetylated by cellular esterases and later oxidized by ROS into a highly fluorescent compound (DCF) which can be quantified by fluorescence (λ_ex_ = 495 nm, λ_em_ = 529 nm).

Sterile ZnOMn NPs suspensions were added in each Petri dish at final NP concentrations of 0, 1, 2, 4, and 8 µg/mL and the cells were further incubated for 24 h. The medium with NPs was then removed and the cells were washed with a sterile buffer (1.26 mM CaCl_2_, 5.4 mM KCl, 0.44 mM KH_2_PO_4_, 0.34 mM Na_2_HPO_4_, 3.31 mM NaHCO_3_, 0.5 mM MgCl_2_, 0.41 mM MgSO_4_, 137 mM NaCl, 5.56 mM D-glucose, pH 7.4). H_2_DCFDA was added to the cells (final concentration 2 µM) and further incubated for 40 min. The cells were washed and the fluorescence intensity was measured (Zeiss Observer D2 inverted microscope, Germany, equipped with RatioMaster D-104C Horiba system, USA, Felix Gx acquisition software). The signal was collected from one single cell during 10 s, and the average value was normalized to the cell surface area (Zeiss AxioVision rel 5.9 and Image J). For each NPs concentration, 30 cells were randomly chosen from 3 different Petri dishes.

### Cellular DNA fragmentation

The comet assay is a single cell electrophoresis technique used to measure DNA fragmentation. The cells are incorporated in an Agarose gel, deposited on a microscope slide, lysed, and subjected to electrophoresis. The genetic material from each cell will migrate depending on its degree of fragmentation: smaller DNA fragments will migrate faster^56^. Samples were stained with Propidium Iodide and fluorescence images were recorded (λ_ex_ = 490 nm, λ_em_ = 510 nm) showing a “comet” shaped pattern. The fragmentation degree was quantified by the tail length of the comet (defined as the distance between the geometrical centers of the head and of the comet).

Serial concentrations of NPs (0, 1, 2, 4 and 8 µg/mL) were incubated for 24 h with the cells, then the cells were harvested (Porcine Trypsin 0.5 g/L and 0.2 g/L Na_4_EDTA in 0.9% NaCl, Sigma T4174), the suspensions were centrifuged (5 min, 300 × g) and the pellet resuspended in PBS (137 mM NaCl, 3 mM KCl, 10 mM Na_2_HPO_4_, 1 mM KH_2_PO_4_) at 10^6^ cells/mL. The suspensions were mixed with 1% Low Melting Agarose (Promega V2111, Spain), volume ratio 1:10, and placed on microscope slides pre-coated with 2% Normal Melting Agarose (Promega V3121, Spain). The cells were lysed in a cold lysis buffer (2.5 M NaCl, 100 mM Na_2_EDTA, 10 mM Tris Base, 5 M NaOH, 1% Triton X-100, pH 10) (4 °C, 1 h). The slides were kept at 4 °C in an alkaline electrophoresis buffer (300 mM NaOH, 20 mM Na_2_EDTA, pH 12.5) for 20 min. Electrophoresis was run at 20 V, 300 mA for 20 min. The slides were then soaked in a cold neutralization buffer (0.4 M Tris Base, pH 7.5) for 15 min at 4 °C. The DNA was stained using 5 µg/mL Propidium Iodide for 30 min^56,57^.

For the positive control, cells untreated with NPs were incubated 10 min with 100 µM H_2_O_2_^37^. As negative control, untreated cells were used (0 µg/mL NPs).

Between 150 and 300 randomly captured images of comets were acquired for each NPs concentration and their tail length was measured.

### Statistical analysis for cellular studies

All data were analyzed using a one-way analysis of variance (ANOVA) with Tukey’s multiple comparison tests to determine statistical significance among treatments using Origin 8.6 software. Differences were considered significant for p < 0.05.
